# TKL3 regulates blood-stage fitness, male gamete fertility, and transmission-stage development in *Plasmodium berghei*

**DOI:** 10.1371/journal.ppat.1014582

**Published:** 2026-09-08

**Authors:** Himadri Shukla, Nirdosh, Satish Mishra

**Affiliations:** 1 Division of Molecular Microbiology and Immunology, CSIR-Central Drug Research Institute, Lucknow, India; 2 Academy of Scientific and Innovative Research (AcSIR), Ghaziabad, India; Université de Genève Centre Médical Universitaire: Universite de Geneve Faculte de Medecine, SWITZERLAND

## Abstract

Malaria parasites must undergo complex developmental transitions to complete their life cycle and transmit between vertebrate and mosquito hosts. These transitions are tightly regulated by protein phosphorylation events, yet the specific kinases involved remain poorly characterized. Here, we investigate the role of a previously uncharacterized tyrosine kinase-like protein, TKL3, in the rodent malaria parasite *Plasmodium berghei*. We show that TKL3 is expressed in blood stages, schizonts, gametocytes, zygotes, and ookinetes and localizes to the cytoplasm. Targeted disruption of the TKL3 gene impairs asexual blood-stage growth, male gamete fertility, and consequently compromises ookinete development. Although oocyst numbers are significantly reduced in mosquitoes, the sporozoites that do form are morphologically normal, retain hepatocyte infectivity, and complete liver-stage development. However, TKL3 knockout sporozoites showed delayed blood-stage patency due to reduced asexual replication. These findings identify TKL3 as an important regulator of parasite growth and transmission, providing new insights into kinases involved in the *Plasmodium* life cycle.

## Author summary

Malaria transmission remains a major obstacle to eradication, particularly in settings where vector control and treatment coverage are limited. Transmission-blocking interventions (TBIs) that disrupt parasite development within the mosquito vector offer a promising complementary strategy, yet few molecular targets have been validated across multiple transmission stages. Here, we identify the *Plasmodium berghei* kinase TKL3 as a critical regulator of parasite transmission, required for male gamete fertility, ookinete differentiation, and successful establishment of midgut infection. TKL3 knockout parasites exhibit severe defects across multiple stages, resulting in a substantial reduction in transmission. Our findings establish TKL3 as a key kinase regulating blood-stage fitness and mosquito-stage development, and provide new insights into the molecular mechanisms underlying malaria parasite transmission.

## Introduction

Malaria remains a significant global public health concern, caused by protozoan parasites of the genus *Plasmodium*. Infection begins when sporozoites are injected into the host’s skin during the blood meal of an infected *Anopheles* mosquito. These motile forms rapidly migrate to the liver, where they invade hepatocytes and undergo extensive asexual replication, producing thousands of merozoites [1]. Upon release into the bloodstream, merozoites invade red blood cells (RBCs), initiating the symptomatic asexual blood stage. Within RBCs, the parasite progresses through ring, trophozoite, and schizont stages. Some asexually replicating parasites undergo sexual differentiation, giving rise to male and female gametocytes, which are essential for transmission to the mosquito vector [2]. Following ingestion by a mosquito, gametocytes develop in the mosquito’s midgut. Male gametocyte activation triggers three successive rounds of mitotic DNA replication [3], leading to exflagellation and the formation of eight motile microgametes [4,5]. In contrast, female gametocytes mature without additional DNA replication and round up before exiting the RBC [6]. Fertilisation of female macrogametes by male microgametes results in zygote formation. The zygote differentiates into an ookinete, which traverses the midgut epithelium to form an oocyst. Sporogony within the oocyst ultimately produces sporozoites that migrate to the salivary glands, completing the life cycle [7].

Gametocyte differentiation and the signalling pathways that initiate this critical developmental process remain poorly understood in *Plasmodium*. Multiple molecular players have been implicated in gametogenesis [7]. Environmental cues from the host, importantly, an increase in pH and a decrease in temperature, are key triggers for male gametogenesis, driving processes such as microgamete assembly, exflagellation, and DNA replication [8–11]. A key trigger of gametocyte activation is xanthurenic acid (XA), a metabolite of the tryptophan breakdown pathway [12,13]. XA stimulation results in elevated intracellular cyclic GMP (cGMP) levels, subsequently activating protein kinase G (PKG) and triggering the release of calcium ions (Ca^2+^) from intracellular stores, including the endoplasmic reticulum [14,15]. The surge in cytosolic Ca^2+^ acts as a second messenger, amplifying the activation signal by engaging calcium-dependent protein kinases (CDPKs), thereby facilitating gametogenesis [16]. Despite these advances, the roles of numerous kinases implicated during male gametogenesis remain to be fully characterised.

Protein kinases have emerged as crucial regulators of male gametogenesis in malaria parasites. Among them, PKG plays an essential role in Ca^2+^ mobilization during gametocyte activation [16,17]. This Ca^2+^ release triggers downstream signalling events, including the activation of calcium-dependent protein kinase 4 (CDPK4), which is important for microgamete development. CDPK4 governs three key stages in the transformation from a haploid male gametocyte to the release of eight motile, flagellated microgametes, one of which is axoneme motility [18,19]. This process is regulated by a synergistic interplay between cGMP and Ca^2+^ signaling pathways, mediated through effectors such as PKG and phosphoinositide-specific phospholipase C (PI-PLC). In addition to CDPK4, two other calcium-dependent protein kinases, CDPK1 and CDPK2, have been identified as critical for exflagellation and subsequent transmission to the mosquito vector [20,21]. Apart from CDPKs, the atypical mitogen-activated protein kinase (MAPK), PbMAP2 is a key regulator of male gametogenesis in *P. berghei* [22–24]. *Plasmodium* encodes two MAPK homologues, MAP1 and MAP2 [25–28]. Although PbMAP2-deficient parasites develop normal gametocytes, they exhibit a severe defect in male gametocyte exflagellation, indicating that PbMAP2 is essential for male gamete formation [23,24].

The protein kinase repertoire of *Plasmodium* species includes members of well-characterized kinase families such as CK1 (Casein Kinase 1), CMGC (CDK [cyclin-dependent kinases], MAPK [mitogen-activated protein kinases], GSK3 [glycogen synthase kinase 3] and CLKs [CDK-like kinases]), AGC (PKA [cyclic-adenosine-monophosphate-dependent protein kinase], PKG [cyclic-guanosine-monophosphate-dependent protein kinase], PKC [protein kinase C] and related proteins), and CaMK (calcium/calmodulin-dependent kinases), as well as kinases related to atypical groups like NIMA-related kinases [29]. However, the functional roles of tyrosine kinase-like (TKL) proteins remain largely unexplored. TKLs are defined by their sequence similarity to classical tyrosine kinases, which are absent from the *Plasmodium* kinome, but they exhibit serine/threonine kinase activity [30]. Some TKL family members, such as RAF (rapidly accelerated fibrosarcoma) and MLKs (mixed-lineage kinases), function as MAP3Ks, initiating MAPK signalling cascades involving three kinases: MAP3K (MEKK), MAP2K (MEK), and MAPK. Others, like RIPK and IRAK, are involved in immune signalling, while LIM kinases are implicated in cytoskeletal regulation [31]. The *Plasmodium* genome encodes four TKL proteins, TKL1-TKL4, which belong to an atypical kinase family with no close mammalian orthologues [30,32]. Functional information on this family remains limited and is largely restricted to TKL2 and TKL3 in *P. falciparum*. PfTKL2 possesses kinase activity and is expressed during both asexual and sexual blood stages, where it is exported from infected erythrocytes [33]. PfTKL3 is a multidomain kinase containing MORN motifs, a sterile α-motif (SAM) domain, a putative PEXEL motif, and a C-terminal kinase domain. It is expressed throughout the parasite life cycle, with highest abundance during schizogony, and displays dynamic subcellular localization [30]. Biochemical studies have confirmed its kinase activity, while the SAM domain regulates kinase function and protein oligomerization. Genetic studies, however, have yielded conflicting results regarding TKL3 function. Early reverse genetics suggested an essential role in asexual blood-stage growth [30], whereas conditional knockdown, targeted knockout, and rodent malaria studies demonstrated that TKL3 is dispensable or causes only a mild growth defect [34]. TKL3 has also been implicated in resistance to PKG inhibitors, suggesting a broader role in parasite signaling [34].

Here, we characterize the function of PbTKL3 and demonstrate that this cytosolic protein is required for blood-stage fitness and successful mosquito transmission. Using PbTKL3 knockout parasites, we observed that these parasites produce male gametes with significantly reduced fertility. Consequently, zygote formation was impaired, and the few zygotes that did form displayed defective ookinete differentiation. This developmental arrest led to a dramatic reduction in both the number and size of oocysts, accompanied by poor sporogony and a substantial decrease in salivary gland sporozoites. Our findings demonstrate that PbTKL3 is an important regulator of male gamete fertility and ookinete development, contributing to the overall fitness of *P. berghei*.

## Results

### PbTKL3 is a SAM domain-containing protein that localises to the cytoplasm

**Fig 1:**
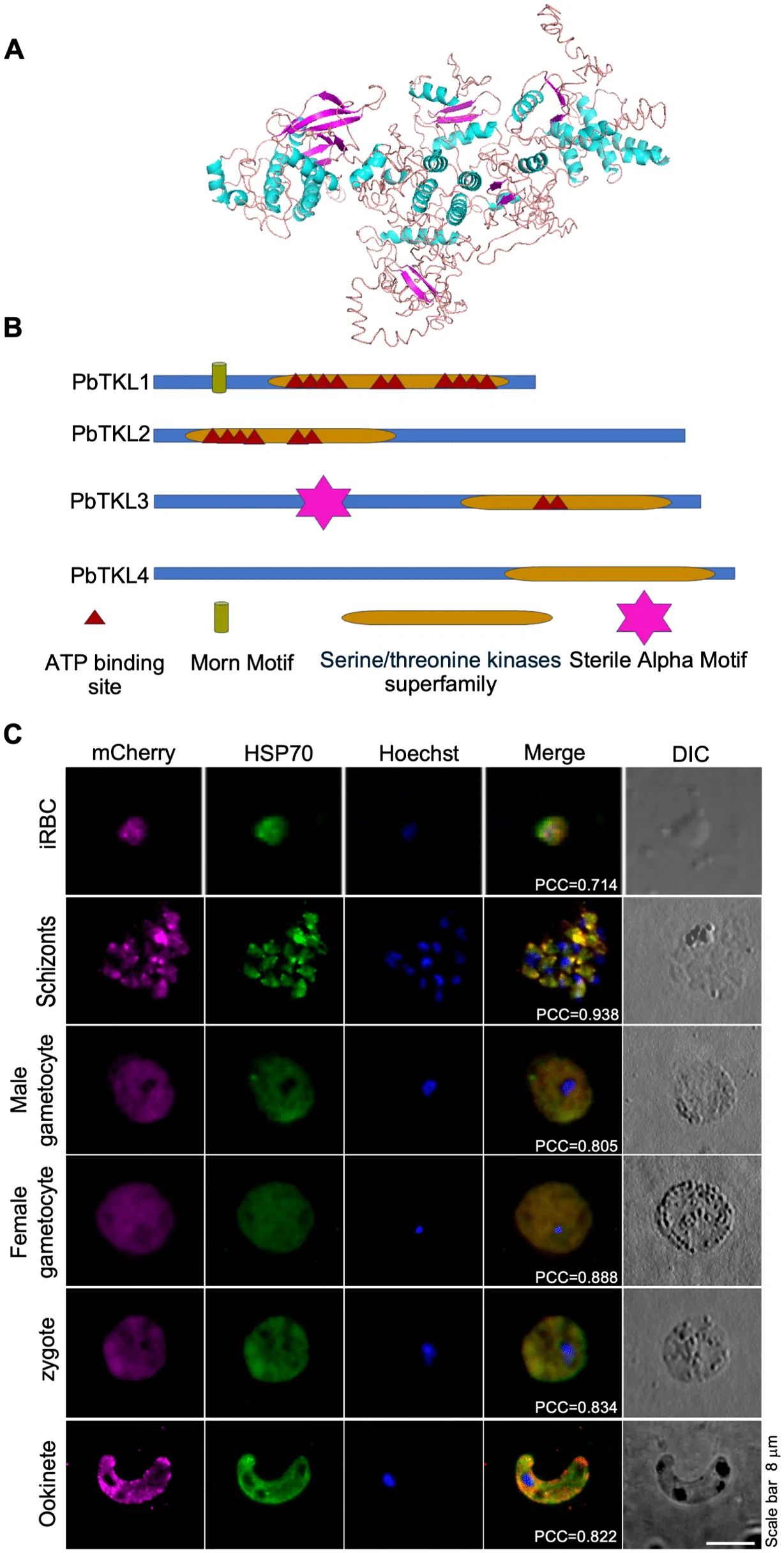
TKL3 is a SAM domain-containing cytoplasmic protein. **(A)** The predicted three-dimensional structure of TKL3 was visualized using PyMOL, revealing a complex fold composed of multiple α-helices (sky blue) and β-sheets (magenta). **(B)** Schematic representation of *P. berghei* TKL1-4 showing the location of the sterile alpha-motif (SAM) domain and the protein kinase domain. Several putative active sites are indicated, including ATP-binding sites located between residues 1033-1041 and at residue 1054. Active sites are highlighted in red, magenta, and yellow on the cartoon model. **(C)** Immunofluorescence assay of iRBCs, schizonts, male and female gametocytes, zygote and ookinetes expressing TKL3 Tr, stained with anti-mCherry and anti-HSP70 antibodies. The mCherry signal colocalizes with the cytoplasmic marker HSP70, supporting cytoplasmic localization. Pearson correlation coefficient (PCC) analysis was performed using the JACoP plugin in Fiji, with five images analyzed per parasite stage.

Phylogenetic analysis of kinase domains identified four TKL family members in *Plasmodium*, designated TKL1-TKL4 [30]. Our phylogenetic analysis showed that *P. berghei* TKL3 is highly conserved across apicomplexan parasites (S1A Fig). Analysis of the SAM domain amino acid sequence revealed strong conservation among *Plasmodium* species (>90% sequence similarity), with moderate conservation in other apicomplexan genera, including *Cystoisospora* (50%), *Besnoitia* (45.55%), and *Eimeria* (44.26%) (S1B Fig). Structural comparison of the AlphaFold-generated TKL3 model using DALI revealed significant structural similarity to several *Plasmodium* kinase-like proteins. Many canonical serine/threonine and other eukaryotic kinases also show significant structural similarity through the conserved ~250–300-residue kinase core. The highest-scoring hit was a *Plasmodium* tyrosine kinase-like protein (Z-score 40.8; 557 aligned residues; 50% sequence identity). TKL3 also showed strong structural similarity to calcium-dependent, NIMA-related, Aurora, CDK, MAPK, and other protein kinases, with Z-scores ranging from 19.5 to 28.0 and RMSDs as low as 2.2 Å. These results indicate that TKL3 adopts a conserved protein kinase-like structural architecture and support its classification within the divergent eukaryotic protein kinase superfamily. In silico protein-protein interaction predictions using the STRING database identified putative TKL3 interaction partners, including metal-binding phosphatases, a putative kinase, and a UBX domain-containing protein (S2A and B Figs). Structural analysis identified a conserved serine/threonine kinase domain (residues 1027–1392) and a SAM domain (residues 933–994) in PbTKL3 (Fig 1A and B). Among the four TKL proteins encoded in the *Plasmodium* genome, only TKL3 possesses a SAM domain (Fig 1B), suggesting a potentially specialised regulatory function. The kinase domain harbours several active sites [30], including ATP-binding motifs between residues 1033–1041 and at residue 1054. Cellular localisation predictions indicate that TKL3 is localised to the cytoplasm. Structure-based phylogenetic analyses further place TKL3 in close evolutionary proximity to several key protein kinases and proto-oncogenes (S2C Fig and S1 Table).

To investigate the expression and subcellular localisation of TKL3, we endogenously tagged the gene with a C-terminal 3XHA-mCherry tag (S3A Fig). Correct integration at the genomic locus was verified by diagnostic PCR (S3B Fig). Next, we examined the development of the transgenic parasites across different stages of the parasite life cycle and found that TKL3 tagging did not affect parasite development in either the mammalian host or the mosquito vector (S3C and D Figs). Live imaging revealed mCherry expression in blood-stage parasites, gametocytes, and ookinetes (S3E Fig). Western blot analysis of gametocyte lysates identified a band which  ~ 194 kDa band, consistent with the predicted size of the TKL3–3XHA-mCherry fusion protein (S3F Fig). Co-localisation with the cytoplasmic marker HSP70 in blood-stage parasites, gametocytes, zygotes, and ookinetes demonstrated that TKL3 is cytoplasmically localised during these life cycle stages (Fig 1C). mCherry signals were not detected in sporozoites or liver stages (S3G and H Figs). All detected signals were specific to the mCherry-expressing transgenic parasites, whereas no signal was observed in WT parasites at any developmental stage when stained with the anti-mCherry antibody (S4 Fig). *P. berghei* TKL3 contains a putative PEXEL motif at its C-terminus, suggesting the possibility of protein export into the host erythrocyte. To determine whether TKL3 is exported, we examined the localization of TKL3–3XHA-mCherry in gametocyte infected erythrocytes using anti-TER119 antibody to mark the erythrocyte membrane. TKL3–3XHA-mCherry fluorescence was restricted to the parasite and was not detected in the erythrocyte cytosol, indicating that TKL3 is not exported beyond the parasite boundary (S5A and B Figs). Together, these data show that TKL3 is expressed and localised to the cytoplasm during the blood-stage, gametocyte, zygote, and ookinete stages of the parasite life cycle.

### TKL3 is important for asexual blood-stage propagation

**Fig 2:**
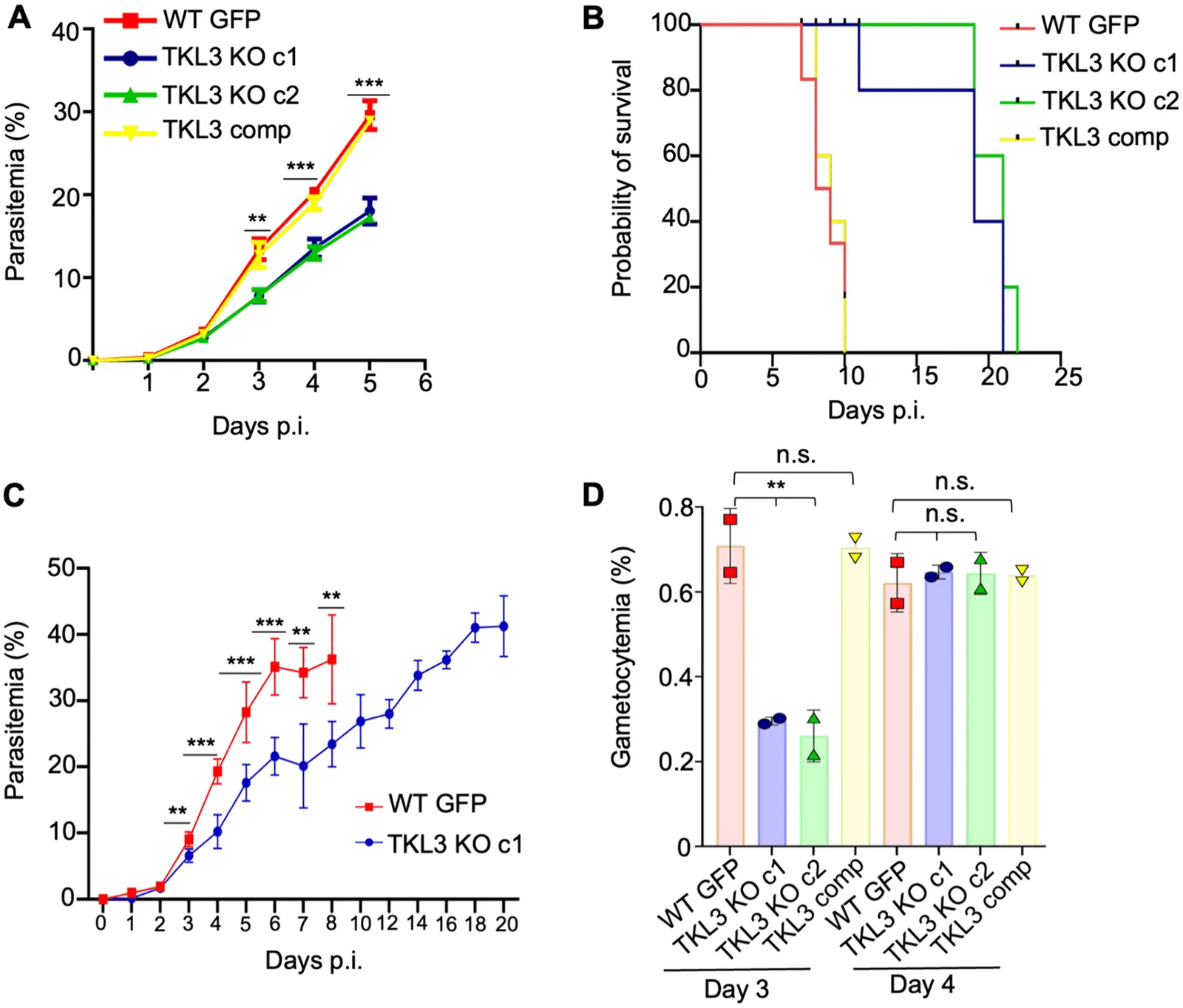
TKL3 is required for intra-erythrocytic development of parasites. **(A)** Equal numbers of control or TKL3 KO parasites were intravenously injected into Swiss albino mice, and parasitemia was monitored by Giemsa-stained blood smears. The growth rate of TKL3 KO parasites was significantly reduced compared to controls. Data represent mean±SEM from three independent experiments (n = 15 mice/group). Statistical significance was assessed by one-way ANOVA (day 1, *P* = 0.6576; day 2, *P* = 0.1739; **P < 0.01; ***P < 0.002). **(B)** Kaplan-Meier survival analysis of mice (n = 10 mice per group) infected with WT GFP, TKL3 KO, or TKL3 comp parasites. **(C)** TKL3 KO parasites showed slower asexual blood growth than WT GFP parasites but exhibited a gradual and sustained increase in parasitemia over the course of infection (n = 5 mice per group; **P < 0.01; ***P < 0.002, unpaired Student’s t-test). **(D)** TKL3 KO parasites showed a significant reduction in gametocyte numbers on day 3 post-infection compared with controls, which returned to levels comparable to controls by day 4. Data represent mean±SEM from two independent experiments (n = 10 mice/group). Statistical significance was assessed by one-way ANOVA (**P < 0.01; day 4*, P* = 0.8641); n.s., not significant.

To investigate the role of TKL3 in the *P. berghei* life cycle, the gene was disrupted via double-crossover homologous recombination (S6A Fig). Recombinant parasites were selected using pyrimethamine drug, and successful transfection was confirmed by GFP fluorescence microscopy (S6B Fig). Deletion of the TKL3 locus was verified by diagnostic PCR (S6C Fig). A complemented parasite line was generated by integrating a TKL3 expression cassette at the TKL3 KO locus (S6D Fig), and successful complementation was confirmed by genotyping (S6E Fig). To assess the effect of TKL3 deletion on asexual blood-stage propagation, Swiss albino mice were intravenously injected with either control or TKL3 KO parasites. Parasite growth rates were monitored by Giemsa-stained blood smears. TKL3 KO parasites exhibited significantly slower asexual growth compared to controls (Fig 2A). All mice infected with WT parasites died by day 10, whereas mice infected with TKL3 KO parasites survived longer, indicating attenuated parasite virulence (Fig 2B). However, prolonged monitoring revealed a gradual but sustained increase in parasitemia in TKL3 KO infected mice (Fig 2C), which likely contributed to their eventual death. Furthermore, gametocyte numbers were significantly reduced in TKL3 KO parasites on day 3 post-infection but returned to normal levels by day 4 (Fig 2D). These findings indicate that TKL3 plays an important role in maintaining parasite fitness during the blood stage of infection.

### TKL3 KO parasites exhibit severe defects in oocyst development and sporozoite formation

**Fig 3:**
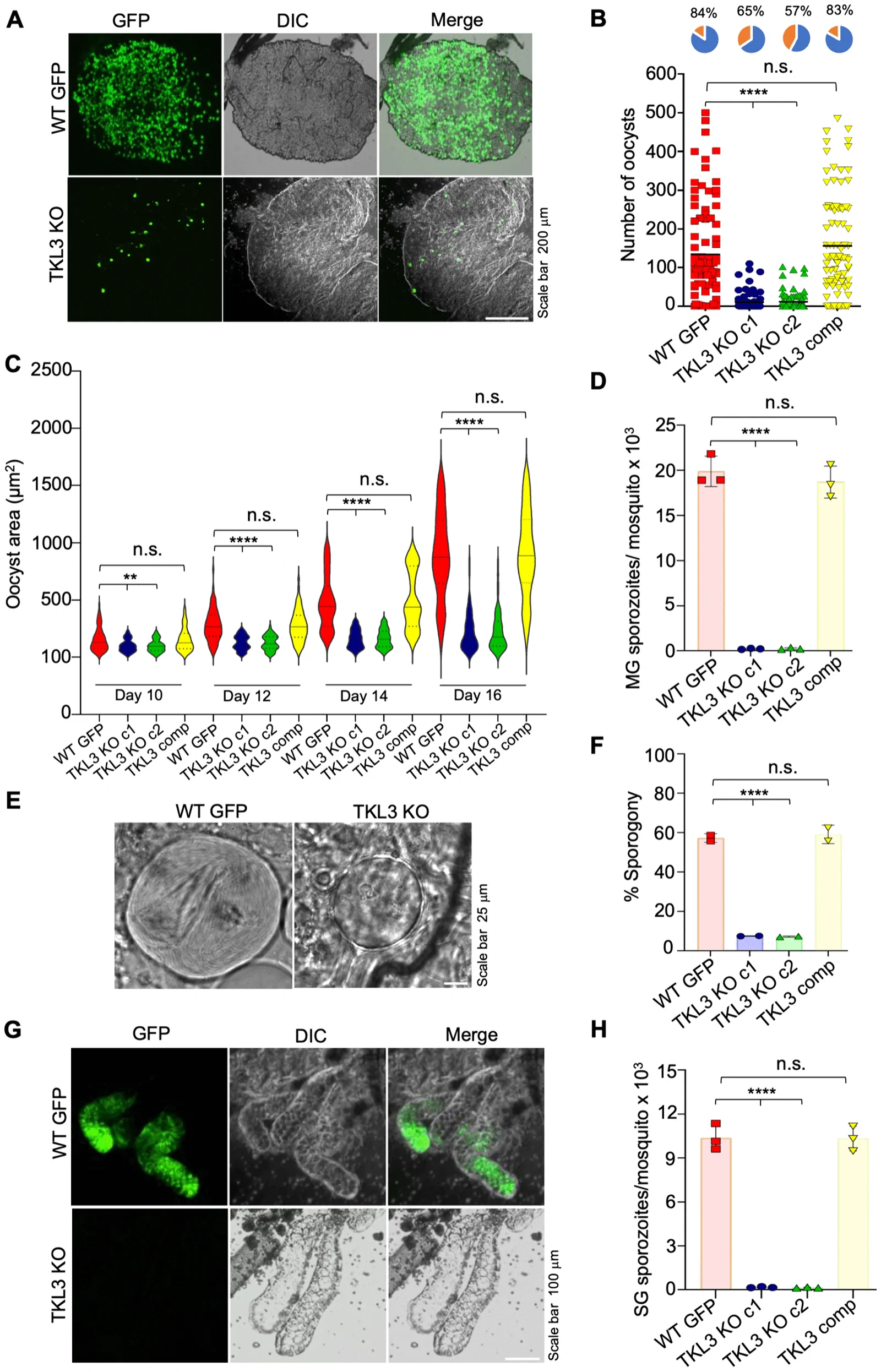
TKL3 is required for oocyst development and sporogony. **(A)** Representative images of mosquito midguts infected with WT GFP or TKL3 KO parasites, showing differences in oocyst development. **(B)** Quantification of oocyst numbers reveals a significant reduction in TKL3 KO parasites compared to WT controls (****P < 0.0001; n = 90 midguts per group pooled from three independent experiments). Oocyst prevalence is presented as a pie chart, with the percentage of oocyst positive mosquitoes indicated for each experimental group. **(C)** Oocyst size measurements from days 10 to 16 post-infection show a significant decrease in TKL3 KO parasites (****P < 0.0001). Complementation of TKL3 restores oocyst size to levels comparable to WT GFP parasites (day 10: P = 0.9020; day 12: P = 0.8597; day 14: P = 0.6191; day 16: P = 0.8446). **(D)** Midgut sporozoite counts are significantly reduced in TKL3 KO parasites relative to controls. Data from three independent experiments presented as mean±SEM (****P < 0.0001). **(E)** Morphological analysis reveals impaired sporogony in TKL3 KO oocysts. **(F)** Quantification of sporogony confirms a significant decrease in TKL3 KO parasites (****P < 0.0001). A total of 593, 212, 159, and 340 oocysts were analysed for WT GFP, TKL3 KO c1, TKL3 KO c2, and TKL3 comp parasites, respectively. **(G)** Due to the very low number of salivary gland sporozoites, GFP fluorescence was not detectable in TKL3 KO parasites. **(H)** Salivary gland sporozoite numbers were significantly reduced in TKL3 KO infected mosquitoes compared to WT (****P < 0.0001). Statistical significance was determined using one-way ANOVA.

To determine the role of TKL3 in malaria transmission, we assessed the development of KO parasites in the mosquito vector. Female *Anopheles* mosquitoes were fed on mice infected with either control or TKL3 KO parasites. Fourteen days after the infectious blood meal, mosquitoes fed on TKL3 KO parasites exhibited a significant reduction in oocyst-level infection intensity, with an average of 18 oocysts per midgut compared with 173 in mosquitoes fed on control parasites. In addition, TKL3 KO parasites showed a 22% reduction in infection prevalence relative to the control group (Fig 3A and B). Quantitative analysis further demonstrated that oocyst size was significantly reduced in TKL3 KO-infected mosquitoes compared with controls (Fig 3C). In line with this developmental defect, TKL3 KO parasites produced significantly fewer midgut sporozoites (Fig 3D), and the small oocysts exhibited impaired sporogony (Figs 3E and F). Furthermore, salivary gland sporozoite numbers were severely reduced in TKL3 KO parasites, and GFP fluorescence associated with sporozoites was not detectable (Figs 3G and H). Importantly, genetic complementation of TKL3 fully restored oocyst development and sporozoite production to WT levels, confirming that the phenotype was specific to TKL3 disruption. These results demonstrate that TKL3 plays an important role in oocyst formation and efficient sporogony in the mosquito host.

### TKL3 is critical for the transition from early to mid-stage ookinete development

**Fig 4:**
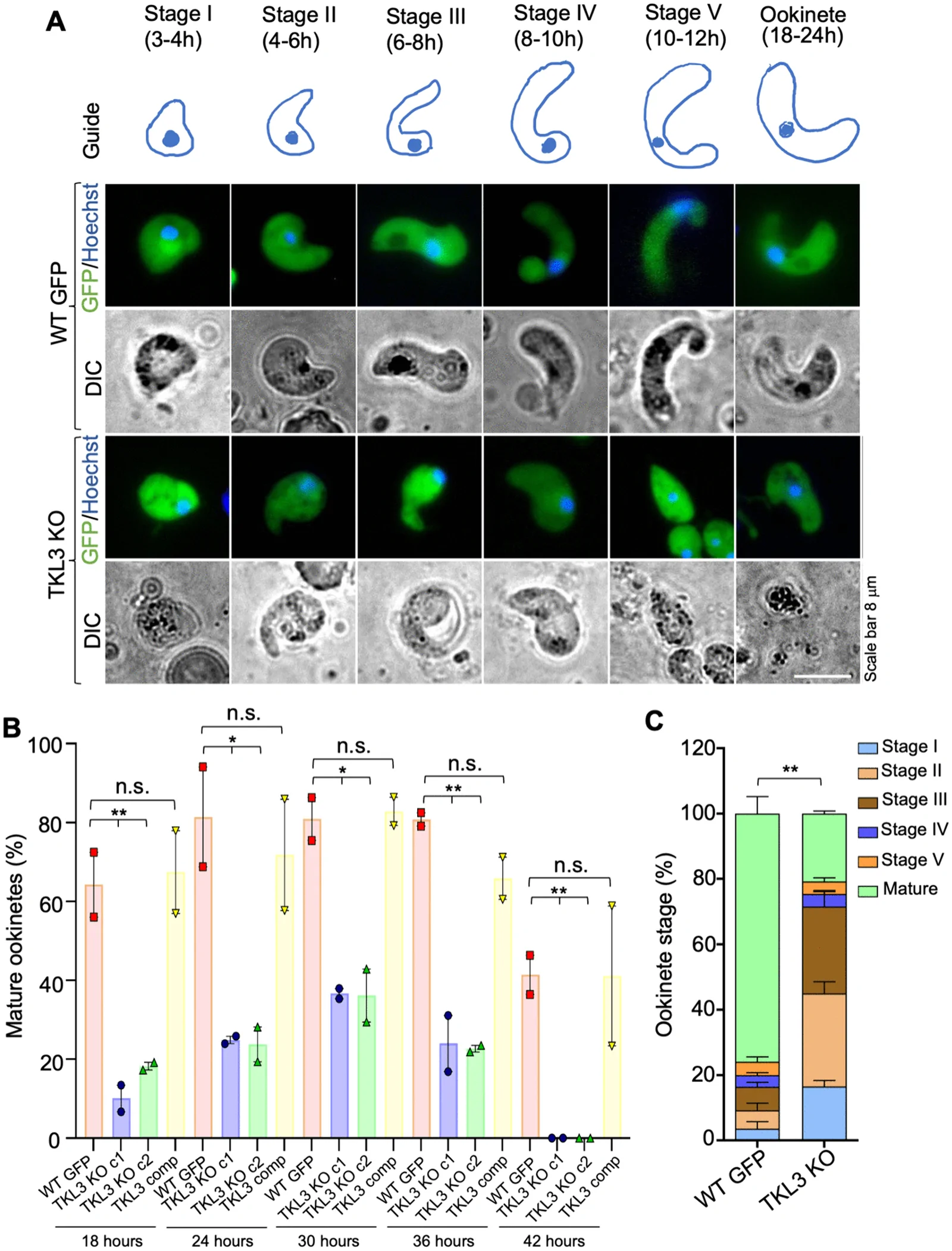
TKL3 is required for ookinete differentiation. **(A)** Live fluorescence microscopy images showing the morphological progression of ookinete development from stage I through intermediate retort stages to mature ookinetes. TKL3 KO parasites exhibit a defect in transitioning from stage II to stage **III. (B)** Quantification of the percentage of mature ookinetes in WT and TKL3 KO parasites. Data represent mean±SEM from two independent experiments. Statistical significance was determined by one-way ANOVA (*P < 0.05, **P < 0.01). **(C)** Percentage of ookinete developmental stages as compared to WT GFP. The proportions of Stage I-III ookinetes were significantly higher, whereas the proportion of mature ookinetes was significantly lower, in TKL3 KO parasites compared to WT GFP. Data represent mean±SEM from two independent experiments with a significant difference (**P < 0.01, Student’s t-test).

To investigate whether the TKL3 KO phenotype results from defects in ookinete differentiation, we collected blood boluses from infected mosquitoes 24 hours post-blood meal and examined them by fluorescence microscopy. Unlike WT parasites, which progressed to mature, elongated ookinetes, TKL3 KO parasites predominantly remained in the retort form, suggesting a developmental arrest. To more precisely define the stage of the block, we conducted a time-course analysis of ookinete development from 3 to 24 hours post-fertilization. WT GFP expressing zygotes advanced through all six stages of differentiation, reaching mature ookinetes (stage VI) by 18–24 hours. In contrast, TKL3 KO parasites were largely arrested between stages II and III, with minimal progression beyond the early retort stage (Figs 4A, B and C). These results demonstrate that TKL3 is required for ookinete maturation and is specifically required for the developmental transition from stage II to stage III.

### TKL3 KO parasites exhibit defects in male gamete fertility

**Fig 5:**
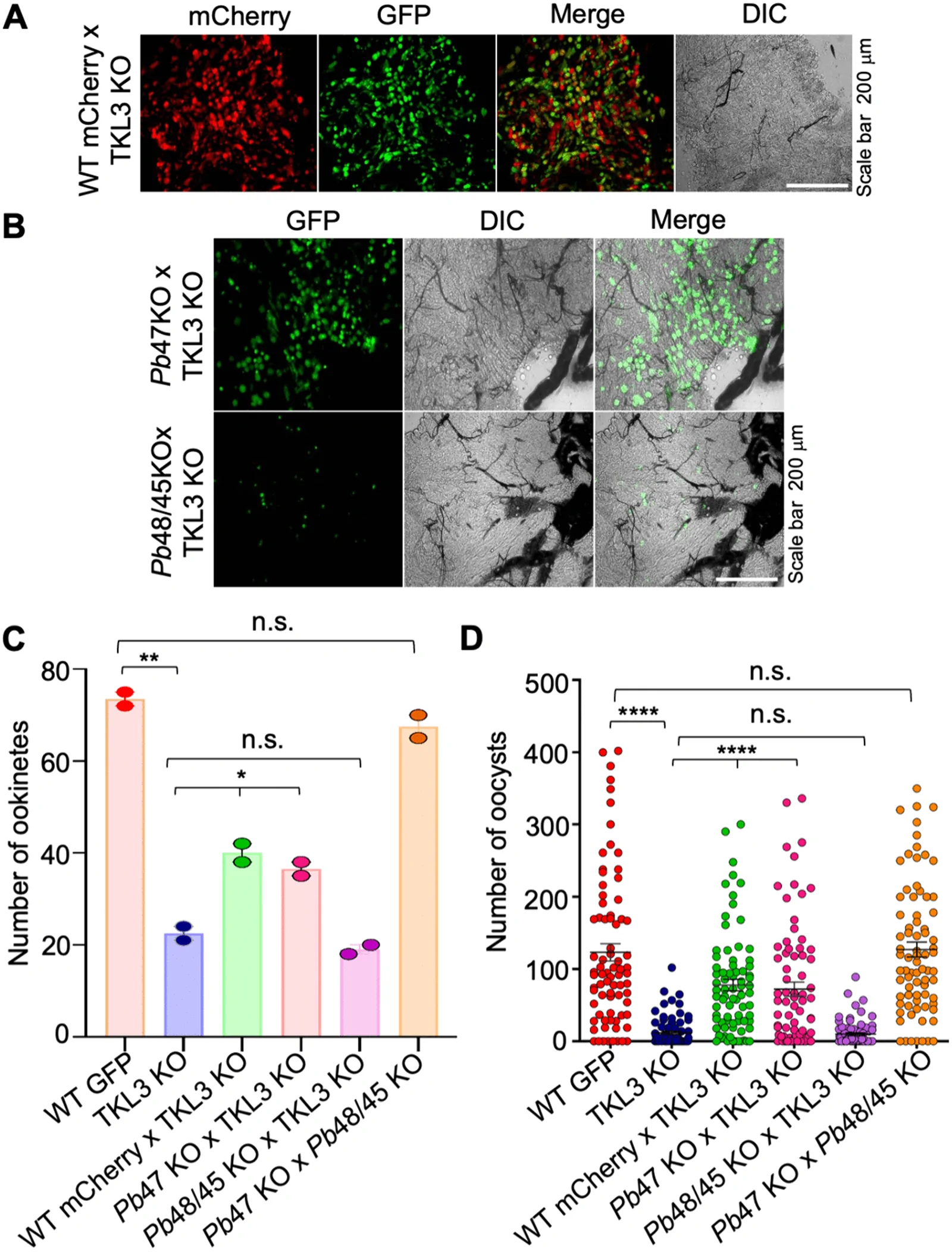
TKL3 is required for male gamete fertility. **(A)** Fluorescence microscopy images depicting oocyst development at day 14 post-blood meal following genetic crosses between WT-mCherry and TKL3 KO parasites. **(B)** The oocyst development defect observed in TKL3 KO parasites was rescued upon genetic crossing with Pb47 KO parasites (female gamete defective), but not with Pb48/45 KO parasites (male gamete defective). **(C)** Quantification of ookinete numbers after genetic crosses. A total of 88 microscopic fields were counted for each group. **(D)** Quantification of oocysts on day 15 post-blood meal after genetic crosses. Results are presented as mean±SEM from two independent experiments. Statistical significance was determined by Student’s t-test or one-way ANOVA (*P < 0.05, **P < 0.01, ****P < 0.0001).

**Fig 6:**
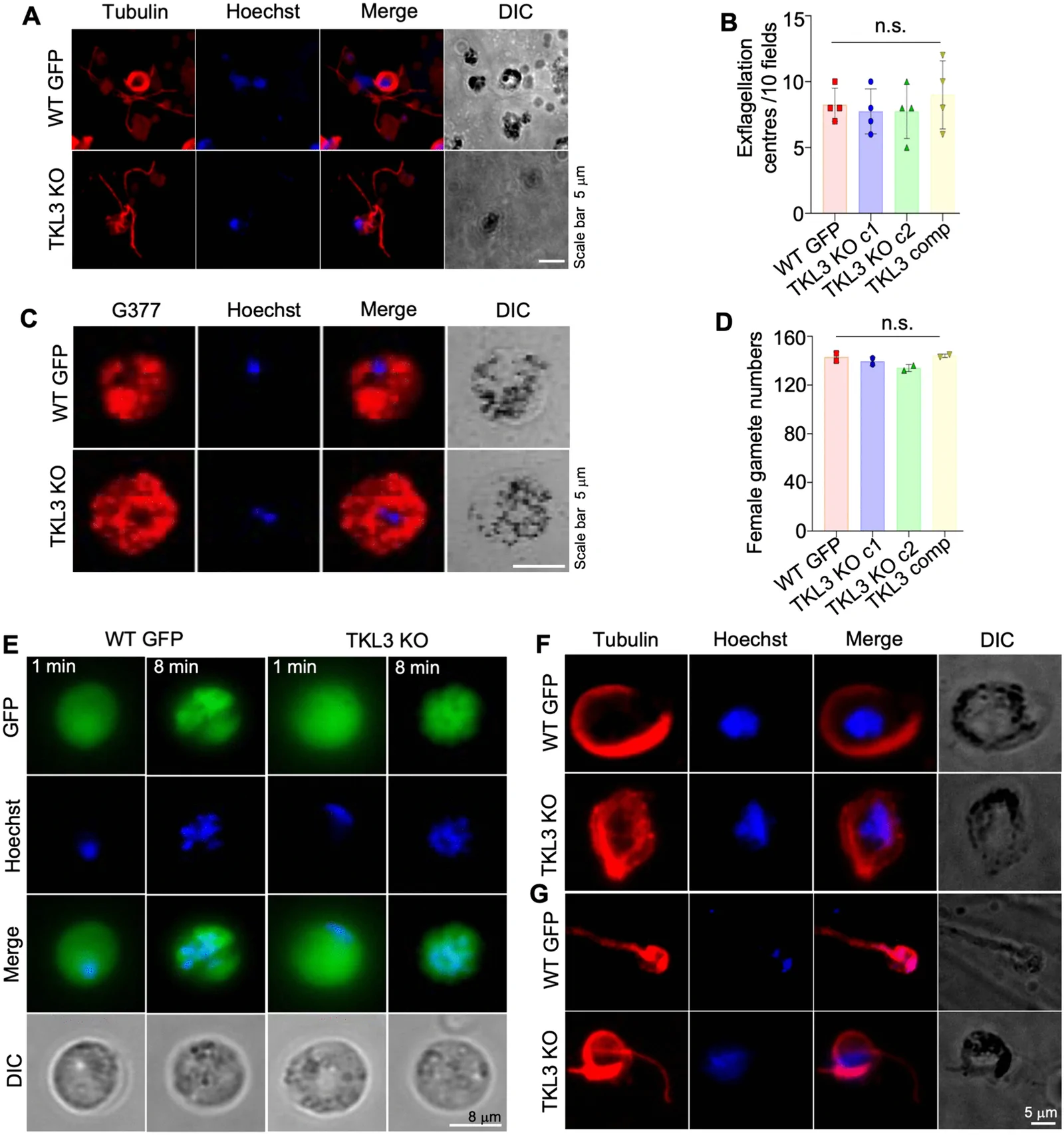
Gametocytes activation in TKL3 KO parasites. **(A)** Male gamete development was assessed by immunofluorescence using an anti-tubulin antibody to visualize exflagellation. **(B)** Quantification of exflagellation centers per 10 microscopic fields showed no significant difference between TKL3 KO and WT GFP parasites. Data from four independent experiments presented as mean±SEM with no significant difference (P = 0.8933). **(C)** Female gametocyte development was visualized with an anti-G377 antibody. **(D)** The number of female gametocyte in TKL3 KO parasites was comparable to WT GFP. Data from two independent experiments presented as mean±SEM with no significant difference (P = 0.1746). **(E)** Live fluorescence microscopy of WT GFP and TKL3 KO activated male gametocytes at 1 and 8 mpa, showing normal DNA replication. **(F and G)** Gametocytes were activated and fixed with paraformaldehyde (PFA) at 8 and 15 mpa. IFA were performed using an anti-tubulin antibody, and DNA was stained with Hoechst.

**Fig 7:**
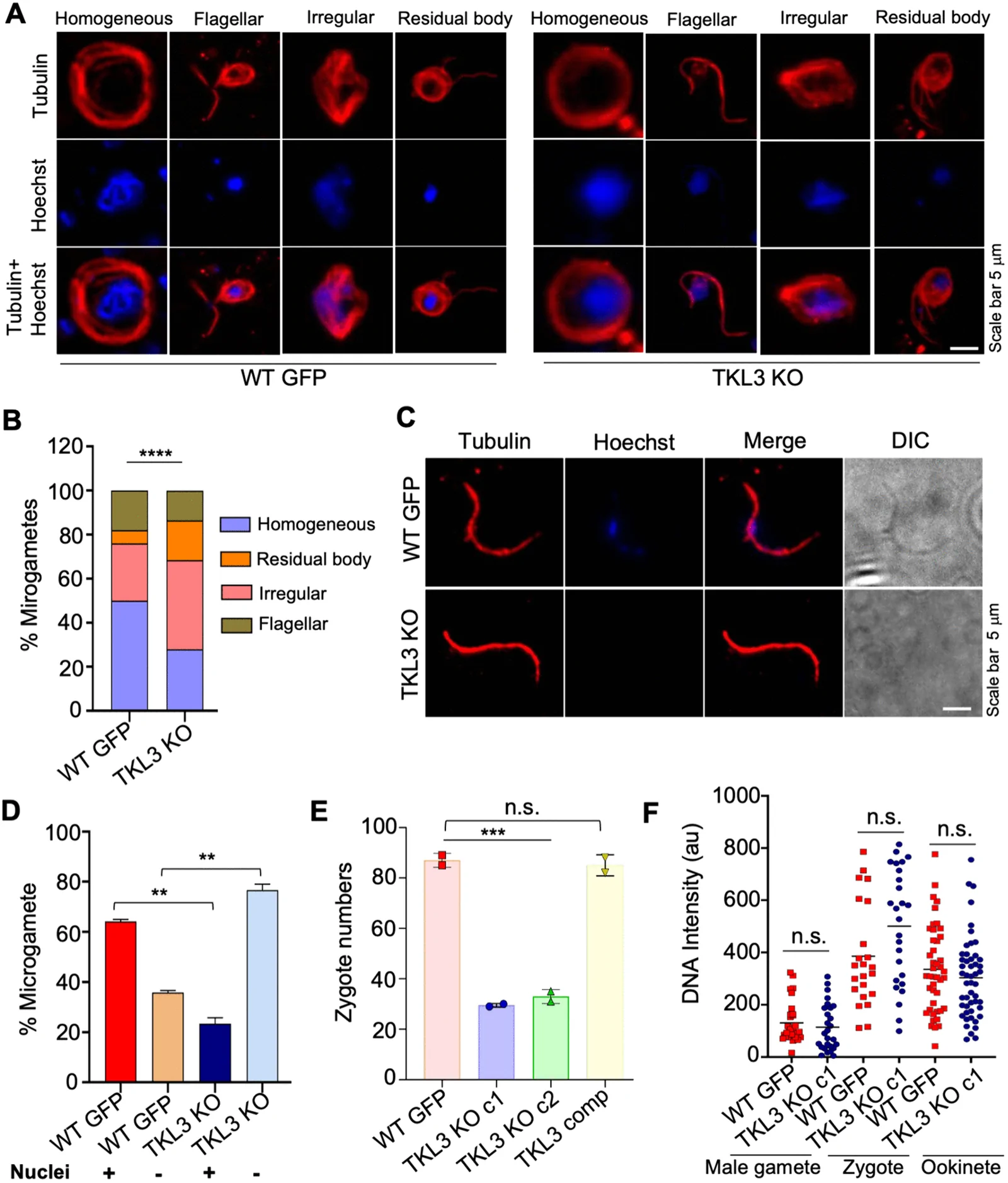
TKL3 KO parasites exhibit defective male gamete function resulting in reduced zygote formation. **(A)** IFA analysis showing DNA distribution patterns in activated microgametocytes of WT and TKL3 KO parasites. **(B)** Quantification of various activated microgametes at 15 mpa based on their DNA localisation pattern. A total of 120 microscopic fields were analyzed for both WT and KO parasites, revealing a significant difference between the two groups (****P < 0.0001, Student’s t-test). **(C)** IFA was performed on WT GFP and TKL3 KO free microgametes using anti-tubulin, and Hoechst was used to stain the parasite nuclei. **(D)** Quantification revealed that approximately 65% of WT gametes contained nuclear DNA, whereas the majority of TKL3 KO microgametes were Hoechst-negative (**P < 0.01, unpaired Student’s t-test). A total of 97 WT GFP and 102 TKL3 KO microgametes were analyzed. **(E)** TKL3 KO parasites produced significantly fewer zygotes compared to controls. Data are presented as mean±SEM from two independent experiments with a significance difference between WT GFP and TKL3 KO (***P < 0.0002, one-way ANOVA). **(F)** Quantification of DNA content in microgametes, zygotes and ookinetes showed no significant differences between TKL3 KO and WT parasites (P = 0.4563, P = 0.0613 and P = 0.3281, respectively, Student’s t-test). A total of 30 male gametes, 25 zygotes and 50 ookinetes were analyzed per group.

To determine whether the block in ookinete maturation observed in TKL3 KO parasites arises from defective gamete function, we performed genetic crosses with WT mCherry parasites or lines deficient in fertile male (Pb48/45 KO) or female (Pb47 KO) gametes [35]. Crosses between TKL3 KO x WT mCherry or TKL3 KO x Pb47 KO parasites resulted in a significant restoration of both ookinete and oocyst numbers, whereas crosses with the TKL3 KO x Pb48/45 KO line failed to rescue these stages (Fig 5A-D), indicating a male-specific defect. To further characterize this phenotype, we isolated gametocytes from infected mice and examined sexual stage development following in vitro gametocyte activation. At 10–20 minutes post-activation, the morphology and numbers of both male and female gametes in TKL3 KO parasites were comparable to those of WT GFP parasites (Figs 6A-D). To assess axoneme formation and DNA replication, TKL3 KO gametocytes were imaged at 1 and 8 minutes post-activation (mpa). IFA using anti-α-tubulin antibody was used to visualize axoneme assembly. By 8 mpa, TKL3 KO gametocytes exhibited robust axoneme formation and DNA replication comparable to WT parasites (Figs 6E, F and G). Despite normal male gametocyte activation, TKL3 KO parasites exhibited defects in DNA distribution during microgametogenesis. In WT parasites, DNA was distributed among distinct classes, including homogeneous, residual, irregular, and flagellar patterns. In contrast, DNA in TKL3 KO parasites was predominantly residual and irregularly distributed (Figs 7A and B). Importantly, the proportion of cells displaying irregular DNA distribution was significantly higher in TKL3 KO parasites compared with WT (Fig 7B). We next analyzed free microgametes by IFA using an anti-tubulin antibody and Hoechst. While microgamete formation in TKL3 KO parasites was comparable to WT, the majority of TKL3 KO microgametes lacked detectable Hoechst staining, indicating an absence of nuclear DNA (Figs 7C and D). Consistent with this observation, zygote formation was significantly reduced in TKL3 KO parasites (Fig 7E), suggesting a failure in successful fertilization. Because male gametogenesis involves three rounds of DNA replication (from 1N to 8N), we quantified DNA content in microgamete, zygote and ookinete. Although most microgametes lacked detectable DNA, the DNA-positive microgametes, zygotes and ookinetes from TKL3 KO parasites exhibited DNA content comparable to that of WT parasites (Fig. 7F). Collectively, these data indicate that although TKL3 KO male gametes undergo normal activation, axoneme assembly, and DNA replication, they display defective DNA allocation, thereby impairing zygote formation efficiency.

### TKL3 is dispensable for parasite liver stage development

To investigate the role of TKL3 in liver stage development, we inoculated C57BL/6 mice with salivary gland sporozoites and monitored the appearance of blood-stage parasites via Giemsa-stained blood smears. Mice infected with TKL3 KO sporozoites consistently exhibited a delayed prepatent period compared to controls (Table 1). To determine whether this delay was due to a defect in liver stage development or reduced blood-stage replication, we performed in vitro assays using HepG2 cells. Invasion assays at 2 hours post-infection (hpi) revealed that TKL3 KO sporozoites entered HepG2 cells at rates comparable to WT GFP parasites (S7A Fig). Assessment of exoerythrocytic forms (EEFs) at 40 and 65 hpi using anti-UIS4 and anti-MSP1 antibodies showed no significant differences in the number, size, or morphology of liver-stage parasites between TKL3 KO and WT GFP (S7B-E Fig). Furthermore, formation of merozoites appeared unaffected by the absence of TKL3 (Fig S7F). Together, these findings suggest that the observed delay in blood-stage patency in vivo is not due to impaired liver stage development, but rather a reduced rate of blood stage parasite proliferation (Fig S7G).

**Table 1:** Infection of TKL3 KO sporozoites in C57BL/6 mice. C57BL/6 mice were intravenously inoculated with WT GFP, TKL3 KO, or TKL3 comp sporozoites. Parasitemia was monitored by examining Giemsa-stained blood smears.

Exp.	Parasite	Number of sporozoites inoculated	Mice positive/Mice inoculated	Prepatent period (days)
**1**	WT GFP	5,000	5/5	3.5
	TKL3 KO c1	5,000	5/5	5.6
	TKL3 KO c2	5,000	5/5	5.4
	TKL3 comp	5,000	5/5	3
**2**	WT GFP	5,000	5/5	3.3
	TKL3 KO c1	5,000	5/5	5
	TKL3 KO c2	5,000	5/5	5.6
	TKL3 comp	5,000	5/5	3.6

## Discussion

**Fig 8:**
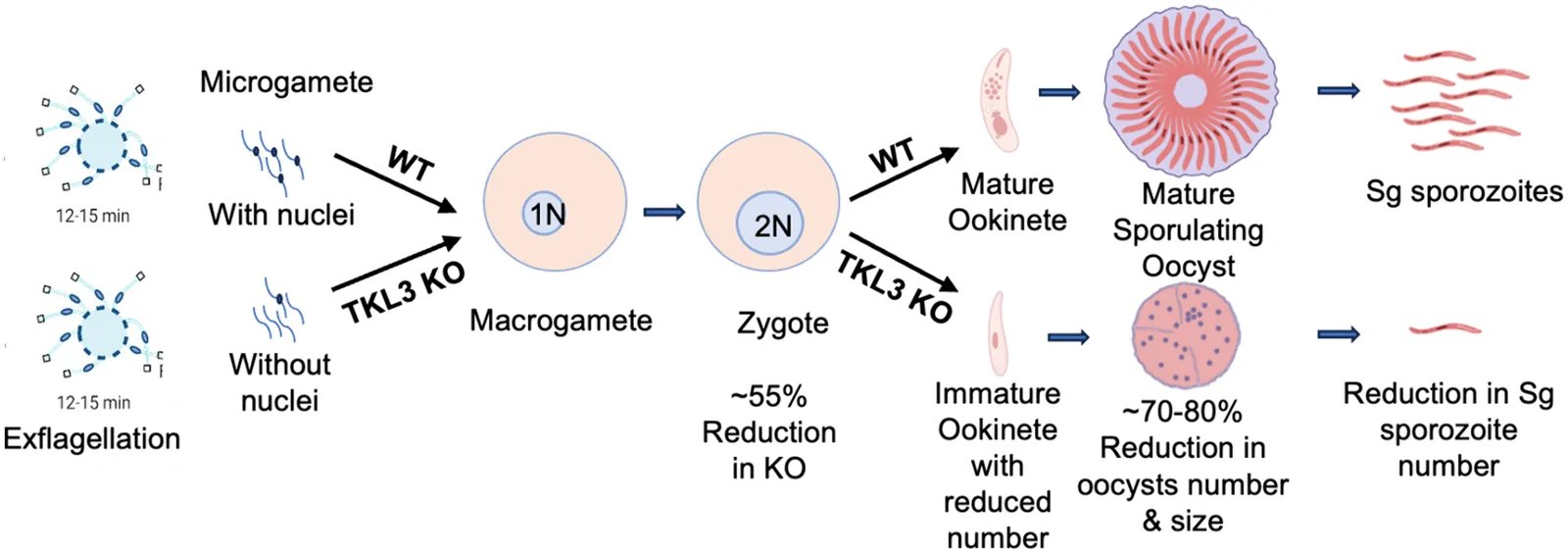
Model illustrating defects in TKL3 knockout parasites. Parasites lacking TKL3 undergo normal exflagellation but exhibit defective DNA segregation in male gametes. Impaired genomic distribution in the male lineage leads to reduced zygote formation and results in aberrant ookinete development, as well as compromised oocyst development and maturation. Created with BioRender.com.

The life cycle of malaria parasites includes asexual proliferation in the mammalian host and the development of gametocytes, which are essential for initiating sexual reproduction in the mosquito vector. Upon ingestion by the mosquito, gametocytes rapidly activate and differentiate into male and female gametes, which fuse to form zygotes. The zygote then differentiates into a motile ookinete, which penetrates the mosquito midgut wall and develops into a thick-walled oocyst. Within the oocyst, the parasite undergoes sporogony, producing thousands of sporozoites that can be transmitted to a new host. Gametogenesis and ookinete differentiation are crucial stages that require precise orchestration of various signalling cascades. *Plasmodium* kinases and ApiAP2 transcription factors are known to regulate gametogenesis and ookinete differentiation [36–38]. However, the roles of *Plasmodium* TKLs remain poorly understood. Here, we characterize the *P. berghei* TKL3 and show that it is an important regulator of blood-stage proliferation, male gamete fertility, ookinete maturation, and sporogony (Fig 8).

Our expression and localization studies show that PbTKL3 is expressed in both blood and mosquito stages and localizes to the cytoplasm. Earlier studies detected PfTKL3 in both asexual and gametocyte stages, where it co-localized with cytoskeletal microtubules in gametocytes, suggesting a role in cytoskeletal organization and cell division [30]. This cytosolic distribution of PbTKL3 is consistent with the localization and functions of several important *Plasmodium* cytosolic kinases, including PfCLK3, PfNek-2, PfNek-4, and PfSRPK1, which regulate erythrocytic development, DNA replication, gametogenesis, and male gamete differentiation [39–42]. PbNEK1 exhibits dynamic spatiotemporal localization, relocalizing from a diffuse cytoplasmic distribution to discrete puncta associated with the bipartite microtubule-organizing center (MTOC), spindle poles, and kinetochores during schizogony and male gametogenesis, where it coordinates spindle assembly, chromosome segregation, and rapid mitosis [43]. PbSRPK1 regulates early male gametogenesis by promoting the first round of DNA replication, while axoneme assembly proceeds normally in octoploid parasites [44]. Together, these observations support a role for TKL3 as a cytoplasmic regulatory kinase involved in parasite and sexual-stage differentiation.

TKL3 is a SAM domain-containing serine/threonine kinase that is specific to apicomplexan parasites [30]. Autophosphorylation and phosphorylation of exogenous substrates by PfTKL3, supports its classification as an active kinase and highlights the importance of the SAM domain for catalytic function [30]. TKL3 has also been implicated in mediating resistance to PKG inhibitors, further suggesting a role in signaling regulation rather than conventional catalysis [34]. In additions to these observations, our genetic analyses establish that TKL3 perform important functions during both asexual blood-stage growth and parasite transmission. Importantly, although TKL3 KO parasites displayed normal gametocyte activation, DNA replication, and exflagellation, male gametes exhibited a profound defect in DNA allocation, indicating that TKL3 functions during post-exflagellation events required for successful zygote formation. PfTKL3 possesses intrinsic kinase activity, a conserved ATP-binding motif, and the ability to phosphorylate exogenous substrates, with both kinase activity and substrate phosphorylation dependent on the SAM domain [30]. These findings suggest that TKL3 functions as a signaling hub, coordinating phosphorylation-dependent pathways required for accurate DNA distribution during male gamete maturation and subsequent transmission-stage development. However, the respective contributions of the kinase and SAM domains to these processes remain unclear and warrant further investigation.

Functional studies on TKL3 have produced contrasting observations regarding its role during erythrocytic development. Initial reverse genetic analyses in *P. falciparum* suggested that PfTKL3 is essential for asexual proliferation, as repeated gene disruption attempts were unsuccessful. In contrast, high-throughput genetic screens in *P. berghei* reported PbTKL3 to be dispensable [45] or associated only with a mild slow-growth phenotype [46]. Similarly, conditional knockdown of PfTKL3 resulted in only a minor growth defect, and successful generation of PfTKL3 KO parasites further demonstrated that TKL3 is not essential for parasite survival [34]. However, our findings demonstrate that PbTKL3 KO parasites exhibit impaired asexual replication, supporting an important and likely conserved role during erythrocytic schizogony. These discrepancies may reflect differences in experimental approaches and the limitations of pooled genetic screening strategies. In particular, earlier PlasmoGEM-based studies raised concerns regarding incomplete homozygous gene disruption. Consistent with this, a similar partial expression pattern of the pseudo-tyrosine kinase-like protein (PbpTKL) was observed using the PlasmoGEM pTKL construct [46]. This limitation was later addressed using a CRISPR-mediated homing strategy to generate homozygous loss-of-function mutants [47].

Consistent with our observations, previous studies have demonstrated that successful zygote-to-ookinete differentiation depends on palmitoylation of the N-terminal cysteines of the IMC proteins ISP1 and ISP3 [48]. In addition, defects in the stage I–II transition during zygote-to-ookinete differentiation have been reported in Nedd8 mutants [49], highlighting the importance of post-translational regulatory pathways during early mosquito-stage development. TKL3 knockout parasites similarly initiate protrusion and early elongation but a significant population fail to complete maturation into fully differentiated ookinetes, suggesting that TKL3 may contribute to signaling pathways that regulate IMC-associated processes and Nedd8-dependent functions during post-fertilization development. Such a role is consistent with the broader requirement for tightly coordinated regulation of cell division, cytoskeletal remodelling, and morphogenesis during parasite differentiation and ookinete maturation. Similarly, the NIMA-related kinases PfNek-2 and PfNek-4 are essential for ookinete formation; parasites lacking these kinases fail to initiate pre-meiotic DNA replication and are arrested prior to full differentiation [39,40]. Recent work further demonstrated that PbNEK4 orchestrates meiotic entry by regulating MTOC duplication, microtubule organization, nuclear migration, and phosphorylation networks required for chromosome condensation and zygote-to-ookinete differentiation, highlighting its central role in parasite transmission [50]. Strikingly, TKL3-deficient parasites show severe defects during sporogony, including reduced oocyst size, number, and sporozoite formation. These defects are unlikely to be solely due to impaired ookinete development, supporting a direct role for TKL3 in oocyst maturation. The phenotype of TKL3 KO parasites is consistent with a role in regulating cytoskeletal organization during parasite development. PfTKL3 localizes to thread-like structures that closely associate with α-tubulin and the subpellicular membrane complex in developing gametocytes, suggesting a function in linking membrane structures to the microtubule cytoskeleton [30]. Its conserved MORN motifs are predicted to mediate membrane interactions, whereas the SAM domain promotes oligomerization and kinase activation, providing a structural framework for organizing signaling complexes. These observations support the hypothesis that TKL3 coordinates cytoskeletal remodeling rather than acting solely as a conventional signaling kinase.

The dispensability of TKL3 during liver-stage development, despite its critical roles during blood and mosquito stages, highlights the stage-specific nature of kinase signaling in *Plasmodium*. The absence of detectable TKL3 expression during hepatic stages suggests that this kinase is not required for liver-stage development. Similar stage-restricted expression patterns and function have been described for proteins such as UIS3 and UIS4, which are highly expressed in sporozoites and liver stages but are largely absent from other stages [51,52]. This pattern suggests that the parasite uses coordinated gene regulation to meet the specific requirements of each life cycle stage. It also raises the possibility that other kinases may compensate for the absence of TKL3 during liver stage development.

Collectively, our findings position TKL3 as an important, multi-functional kinase that integrates cytoplasmic signaling during parasite development and transmission. The presence of a SAM domain and predicted interactions with phosphatases and UBX domain proteins further suggest a role for TKL3 in assembling or modulating signaling complexes, a hypothesis that warrants future biochemical validation. Future studies should focus on identifying TKL3 substrates, dissecting SAM domain interactions, and resolving its stage-specific activation mechanisms.

## Methods

### Ethics statement

All animal procedures were performed in accordance with the guidelines approved by the Institutional Animal Ethics Committee (IAEC) of the Council of Scientific and Industrial Research-Central Drug Research Institute (CSIR-CDRI), under approval numbers IAEC/2018/03 and IAEC/2025/33.

### Experimental organisms, parasites, and cell lines

Female Swiss albino and C57BL/6 mice, aged 6–8 weeks, were bred and housed at the CSIR-CDRI animal facility. These mice were used to study the life cycle of *P. berghei*, including parasite propagation, transmission, and sporozoite infection. The *P. berghei* ANKA (MRA-311) and *P. berghei* ANKA GFP (MRA-867, 507 m6cl1) parasite strains were sourced from BEI Resources (USA). *Anopheles stephensi* mosquitoes were reared in the CSIR-CDRI insectary at 28°C and 80% relative humidity, with access to a 10% sucrose solution. Female mosquitoes were infected with *P. berghei* sporozoites as previously described [53]. For in vitro studies, human hepatocellular carcinoma (HepG2) cells were utilized to assess sporozoite infection and EEF development, as previously described [54].

### In silico characterization of the *P. berghei* TKL3 protein

The amino acid sequence of *P. berghei* TKL3 protein was retrieved from PlasmoDB (PBANKA_1362100) for in silico characterization. Multiple sequence alignment and phylogenetic analysis were conducted using ClustalW implemented in MEGA11 software, and the phylogenetic tree was constructed using the Maximum- Likelihood method with 500 bootstrap to assess the branch distance. The resultant phylogenies were visualized through the Interactive Tree of Life (iTOL) server [55]. Sequence conservation across related proteins was evaluated by generating a protein sequence similarity matrix utilizing the UniProt alignment tool. To investigate potential protein-protein interactions within the parasite, the STRING database was employed. Due to the lack of an experimentally resolved structure for TKL3 in the Protein Data Bank (PDB) and low sequence homology to existing PDB entries, the three-dimensional (3D) structure of TKL3 was predicted using I-TASSER [56]. The predicted model was subsequently visualized and analyzed for conformational features using PyMOL [57]. Functional domain analysis and active site prediction were performed using the NCBI Conserved Domain Database (CDD) server. Subcellular localization of the TKL3 protein was inferred using DeepLoc-2.1 [58]. To further explore evolutionary relationships and structural similarities with other protein kinases, the AlphaFold3-predicted 3D structure of PbTKL3 was analysed for structural similarity using the DALI server. The predicted TKL3 structure served as the query in the DALI database to identify proteins with significant structural similarity. Homologs were ranked by DALI Z-scores, and factors such as the number of aligned residues, RMSD, sequence identity, and functional annotations were also examined [59].

### Generation of TKL3–3XHA-mCherry transgenic parasites

To generate a transgenic *P. berghei* line expressing TKL3 tagged with 3XHA-mCherry, we employed double-crossover homologous recombination as previously described [60]. Two homologous genomic fragments flanking the target locus (F1: 0.46 kb; F2: 0.5 kb) were amplified using primer pairs 2053/2054 and 2055/2056, respectively. These fragments were cloned into the pBC-3XHA-mCherry-hDHFR vector at the XhoI/BglII (F1) and NotI/AscI (F2) restriction sites. The resulting plasmid was linearized with XhoI and AscI and transfected into *P. berghei* ANKA schizonts following established protocols [61]. Drug-resistant parasites were selected via oral administration of pyrimethamine [62]. Integration of the transgene was confirmed by PCR using primer sets 2071/1392 and 1215/1626, and expression of the mCherry fusion protein was verified by fluorescence microscopy. For localization and expression analysis, transgenic parasites (TKL3 Tr) were transmitted to *Anopheles* mosquitoes through blood feeding on infected mice, and parasite stages were examined by immunofluorescence assay (IFA) on prepared slides as previously described [63].

### Western blotting

Blood-stage parasite lysates expressing the TKL3–3XHA-mCherry fusion protein were prepared as previously described [64]. Proteins were separated by electrophoresis on a 10% SDS-PAGE gel and transferred onto nitrocellulose membranes (Bio-Rad). Membranes were blocked in 1% bovine serum albumin (BSA) in phosphate-buffered saline (PBS) for 1 hour at room temperature, followed by overnight incubation at 4°C with an anti-mCherry antibody (1:1,000; 1C51, ThermoFisher) diluted in 1% BSA in PBS containing 0.1% Tween-20 (PBST). After three washes with PBST, membranes were incubated with horseradish peroxidase (HRP)-conjugated anti-rabbit IgG secondary antibody (1:5,000; Amersham Bioscience) for 1 hour at room temperature. Following additional washes, protein bands were visualized using enhanced chemiluminescence substrate (ECL; Bio-Rad) and imaged with a ChemiDoc XRS+ System (Bio-Rad). Membranes were subsequently stripped and reprobed with anti-β-actin antibody (1:500; Sigma Aldrich, A3854) as a loading control.

### Generation of TKL3 KO and complemented parasite lines

To disrupt the *P. berghei* TKL3 gene, two DNA fragments corresponding to the 5’ and 3’ untranslated regions (UTRs) were amplified by PCR. The 5’ UTR fragment (0.61 kb) was amplified using primers 1583/1584, and the 3’ UTR fragment (0.51 kb) with primers 1585/1586. These fragments were cloned into the pBC-GFP-hDHFR:yFCU vector at the XhoI/SalI (5’ UTR) and NotI/AscI (3’ UTR) restriction sites, respectively. The resulting construct was linearized with XhoI and AscI and transfected into purified *P. berghei* ANKA schizonts as described previously [61]. Transfected parasites were selected using pyrimethamine, and drug-resistant, GFP-expressing parasites were cloned by limiting dilution. Successful 5’ and 3’ integration and disruption of the TKL3 open reading frame (ORF) were confirmed by diagnostic PCR using primer pairs 1625/1225, 1215/1626, and 1652/1626, respectively. To generate a complemented parasite line, a fragment encompassing the entire TKL3 ORF together with its native 5’ and 3’ UTRs was amplified using primers 1583 and 1586. This fragment was transfected into the TKL3 KO parasites, and selection was performed using 5-fluorocytosine (5-FC) as described previously [65]. Restoration of the TKL3 locus was confirmed by PCR using primers 1652 and 2444, and clonal complemented lines were obtained by limiting dilution. Two independent TKL3 KO clones derived from separate transfections were used for subsequent phenotypic analyses. Primer sequences are provided in S2 Table.

### Gametocyte culture and activation assay

For the analysis of exflagellation centres, female gamete and zygote, a gametocyte activation assay was performed as described previously [49]. Briefly, two groups of Swiss albino mice were intravenously injected with 200 μl of WT GFP and TKL3 KO *parasites* infected red blood cells (iRBCs) at 0.5% parasitemia. Following the development of high parasitemia, blood samples were collected via cardiac puncture and diluted in RPMI 1640 medium. For zygote counting, parasites were purified using a 50% Nycodenz density gradient centrifugation. iRBCs containing gametocytes, either before or after purification, were activated by incubation in activation medium (25 mM HEPES, 4 mM sodium carbonate, 1 μg/ml neomycin, 10% heat-inactivated fetal bovine serum, 100 µM xanthurenic acid (pH 8.0) at 19°C for 15 minutes. Ex-flagellation centers were enumerated across 10 fields using a Leica DM 3000 LED microscope under a 100x oil immersion objective (NA 1.25). Slides containing various parasite developmental stages were prepared and fixed for subsequent IFA. To assess gamete DNA content, fluorescence intensity of gametes was quantified using Fiji software by measuring total fluorescence and subtracting background signal.

### Analysis of mutant phenotype in blood and mosquito stages

To evaluate the asexual blood-stage propagation of mutant parasites, two groups of Swiss albino mice (n = 5 per group) were intravenously injected with equal numbers of infected red blood cells (iRBCs) harboring either WT GFP or TKL3 KO parasites. Parasitemia was monitored via Giemsa-stained thin blood smears. For analysis of mosquito-stage development, female *Anopheles* mosquitoes were fed on infected mice and dissected at different time points post-blood meal. Ookinete differentiation was evaluated 19–24 hours post-blood meal by dissecting midguts and counting ookinetes using a hemocytometer, as previously described [49]. Oocyst development, sporogony, and sporozoite transmission to salivary glands were assessed on days 14 and 18 post-blood meal, respectively, as previously described [66].

### Genetic cross experiments

To determine the defect at the gamete level, the TKL3 KO parasite was crossed with male (Pb48/45 KO) or female (Pb47 KO) gamete-defective parasite lines, as previously described [35]. We performed genetic crosses by infecting mice with a mixture of blood containing both Pb48/45 KO or Pb47 KO parasites and TKL3 KO parasites. Subsequently, mosquitoes were allowed to feed on these infected mice to initiate infection. For oocyst quantification, GFP-positive oocysts were first counted by fluorescence microscopy. The same mosquito midguts were then stained with 0.5% mercurochrome to visualize non-fluorescent oocysts produced by the gamete-defective parasite lines. Briefly, dissected mosquito midguts were incubated in 0.5% mercurochrome for 10 min, washed in PBS for 10 min, and fixed in 4% formaldehyde for 20 min. Oocysts were subsequently enumerated by light microscopy.

### In vivo infectivity of sporozoite

To evaluate the infectivity of sporozoites in vivo, salivary gland sporozoites were isolated and intravenously injected into C57BL/6 mice. Following injection, blood-stage infection was monitored by preparing Giemsa-stained blood smears.

### In vitro EEF development assays

EEF development was quantified in HepG2 cells as previously described [54] with minor modifications. Briefly, 55,000 HepG2 cells per well were seeded onto type-I collagen-coated coverslips in 48-well plates. After 24 hours, 5,000 salivary gland sporozoites were added to each well, and the plates were centrifuged at 320xg for 4 minutes to facilitate sporozoite contact and invasion. To assess sporozoite invasion, cultures were fixed with 4% paraformaldehyde at 2 hpi. For monitoring EEF development, cultures were fixed at 40 and 65 hpi.

### Immunofluorescence assay

Sporozoite infected cultures fixed at 2 hpi were immunostained with anti-CSP antibody [67] before and after permeabilization, as previously described [68]. For localization studies, different developmental stages of TKL3–3XHA-mCherry parasites were fixed in 4% paraformaldehyde. Samples were washed in PBS. Blood and mosquito stage parasites were permeabilized with 0.1% Triton X-100 (Sigma Aldrich, T8787), whereas EEF cultures were permeabilized using 100% chilled methanol. Samples were blocked with 1% BSA in PBS for 1 hour at room temperature (RT), followed by incubation with primary antibodies: anti-mCherry (1:1,000; clone 1C51, Thermo Fisher Scientific), anti-HSP70 (1:1,000) [69] and anti-mouse TER-119 (1:500; BD Biosciences, 51-09082J) for 1 hour at RT. To distinguish male and female gametes, parasites were stained with anti-tubulin (1:100) or anti-G377 (1:100) antibodies [49], respectively. EEFs were additionally immunostained with anti-UIS4 (1:1,000) [52] and anti-MSP1 (1:5,000) [70]. Secondary antibodies conjugated to Alexa Fluor 488 or Alexa Fluor 594 (Invitrogen, 1:1,000) were used to visualize the primary antibody signals. Nuclei were stained with Hoechst 33342 (Sigma Aldrich, 41399), and samples were mounted with ProLong Diamond antifade reagent (Invitrogen, P36970). Imaging was performed on a Leica SP8 confocal microscope using a 100x oil immersion objective, and images were processed with ImageJ software [71].

### Statistical analysis

Graphs were generated and statistical significance was calculated using GraphPad Prism 9 software. Statistical analyses were performed using unpaired Student’s t-test for comparisons between two groups or one-way ANOVA for comparisons among multiple groups.

## Supporting information

S1 Fig (A) Phylogenetic analysis showing that TKL3 is highly conserved across the Apicomplexa phylum. **(B)** UniProt sequence alignment similarity matrix of the TKL3 SAM domain, demonstrating high conservation among *Plasmodium* species (>90%) and moderate conservation in other apicomplexan genera, including Cystoisospora (50%), Besnoitia (45.55%), and Eimeria (44.26%).
(TIF)

S2 Fig (A) Protein-protein interaction network of TKL3 analyzed using the STRING database. Visualization of TKL3 interaction partners and their network connectivity. Nodes represent proteins, and edges indicate predicted or known associations based on STRING data. (B) Table listing proteins predicted to interact with TKL3. (C) Structure-based phylogenetic tree showing the evolutionary relationship of TKL3 with MAP kinase family members and proto-oncogene proteins. Structural alignment suggests shared evolutionary origins and potential functional similarities among these proteins.
(TIF)

S3 Fig Generation and characterization of TKL3–3XHA-mCherry parasites. (A) Schematic representation of the endogenous tagging strategy used to fuse the 3XHA-mCherry to the C-terminus of the *TKL3* gene. The promoter and 3’UTR are indicated by a vertical arrow and lollipop, respectively. Horizontal arrows denote primers used for diagnostic PCR. (B) Diagnostic PCR confirming correct 5’ and 3’ integration using primer pairs 2071/1392 and 1215/1626, respectively. (C) Quantification of salivary gland sporozoite numbers from two independent experiments demonstrates that gene tagging does not affect sporozoite formation (P = 0.6503, unpaired Student’s t-test). (D) In vivo infection in C57BL/6 mice (n = 5 per group) reveals that *TKL3*-tagged sporozoites infect mice with efficiency comparable to WT. (E) Fluorescence microscopy images showing mCherry expression in iRBC, gametocytes and ookinetes, indicating stage-specific expression of the TKL3. (F) Western blot analysis confirming expression of the TKL3–3XHA-mCherry (TKL3 Tr) fusion protein in gametocyte lysates using an anti-mCherry antibody. The fusion protein migrates slightly higher than the predicted molecular weight of ~194 kDa. The blot was subsequently stripped and reprobed with anti-HSP70 antibody as a loading control. (G and H) No detectable mCherry fluorescence was observed in sporozoites or liver-stage parasites.
(TIF)

S4 Fig Lack of mCherry signal in WT parasites. Immunofluorescence analysis of iRBCs, schizonts, gametocytes, and ookinetes from WT parasites stained with anti-mCherry and anti-HSP70 antibodies. No mCherry staining was detected in WT parasites.
(TIF)

S5 Fig TKL3 localization in infected erythrocytes. (A) IFA analysis of TKL3-Tr parasite with anti-TER-119 and anti-mCherry. The mCherry signal was mainly observed inside the TKL3-Tr gametocytes. (B) Quantitative data showed that 88% of parasites had TKL3 exclusively within the parasite cytoplasm, while 12% showed apparent overlap with the RBC membrane and gametocytes, which is likely due to membrane proximity at this stage. The data represent two independent experiments with a significant difference (***P < 0.0002, unpaired Student’s t-test).
(TIF)

S6 Fig Generation of TKL3 KO and complemented parasites. (A) Schematic representation of the gene replacement strategy. The endogenous *TKL3* locus was replaced via double crossover homologous recombination with a cassette containing the *hDHFR:yFCU* selectable marker and a GFP expression cassette. (B) GFP-expressing *TKL3* KO blood-stage parasites. (C) Diagnostic PCR confirming correct 5’ and 3’ integration of the targeting construct at the TKL3 locus using primer pairs 1625/1225 and 1215/1626, respectively. Absence of WT parasites in the KO clonal line was confirmed using primer pair 1652/1626, which amplified a region within the ORF (WT locus) from genomic DNA of WT but not from KO parasites. (D) Schematic of the gene complementation approach. (E) Restoration of *TKL3* gene in complemented parasites confirmed by PCR using primers 1652/2444.
(TIF)

S7 Fig TKL3 is dispensable for liver stage development but affects blood stage propagation. (A) Hepatocytes were infected with WT GFP or TKL3 KO parasites. Cultures were fixed at 2 hpi and immunostained with an anti-CSP antibody before and after permeabilization to assess invasion efficiency. No significant difference was observed between WT GFP and TKL3 KO parasites (P = 0.7815). Data are presented as mean±SEM from two independent experiments. (B and C) Liver stage parasite growth and maturation were assessed by immunostaining with anti-UIS4 and anti-MSP1 antibodies, respectively. Nuclei were counterstained with Hoechst 33342. (D) Quantification of EEFs revealed no significant difference in parasite numbers between WT GFP and TKL3 KO parasites (P = 0.1799). Data were pooled from three independent experiments. (E) Measurement of EEF size at 40 and 65 hpi showed no significant differences between WT GFP and TKL3 KO parasites (P = 0.9488 and P = 0.2343, respectively). (F) Quantification of liver stage developmental forms, including schizonts, cytomeres, and merozoites, indicated no significant differences between WT GFP and TKL3 KO parasites (P = 0.0760, 0.6764, and 0.1024, respectively). (G) In vivo blood stage growth kinetics revealed a significant delay in parasitemia onset in mice infected with TKL3 KO parasites compared to WT, reflecting slower asexual blood stage propagation (****P < 0.0001 from days 6–10 post-infection; 5 mice per group). Data represent two independent experiments analyzed by one-way ANOVA.
(TIF)

S1 Table List of PDB IDs and proteins used for alignment by the DALI server. (PDF)

S2 Table List of primers used in this study. (PDF)

S1 Data Raw data supporting all the quantitative analyses used to generate graphs in the manuscript. Excel sheet comprises data used to generate both main and supplementary figures. Each sheet is labelled according to corresponding figures and panels.
(XLSX)

S1 Raw Gel Original uncropped and unadjusted images used to generate S3B Fig, S3F Fig, S6C Fig, and S6E Fig. Blots and gels are labelled with respective figures and panels.
(PDF)
