# Supplementary material for: TKL3 regulates blood-stage fitness, male gamete fertility, and transmission-stage development in Plasmodium berghei
Source: PLoS Pathog. 2026 Sep 8;22(9):e1014582. doi: 10.1371/journal.ppat.1014582 (PMC13577473; doi:10.1371/journal.ppat.1014582)
Supplement: Supplementary file 1 [file ppat.1014582.s008.pdf]

S1 Table. List of PDB IDs and proteins used for alignment by the DALI server.

PDB ID	Protein name
S001A	QUERY TKL3
4Y93A	NON-SPECIFIC PROTEIN TYROSINE KINASE
7KJCA	EPHRIN TYPE-A RECEPTOR 2
4FL2A	TYROSINE PROTEIN KINASE
8QGYB	MITOGEN-ACTIVATED PROTEIN KINASE KINASE KINASE
5DZCA	CGMP-DEPENDENT PROTEIN KINASE
7MYJC	5'AMP-ACTIVATED PROTEIN KINASE CATALYTIC SUBUNIT
6TY4A	FOCAL ADHESION KINASE 1
8DGTA	SERINE/THREONINE PROTEIN KINASE BRAF
6C9JA	5'AMP-ACTIVATED PROTEIN KINASE CATALYTIC SUBUNIT
3UTOB	TWITCHIN KINASE REGION
8CAVA	SERINE/THREONINE PROTEIN KINASE
8EWYA	TYROSINE PROTEIN KINASE
7NURA	PROTEIN KINASE DOMAIN CONTAINING PROTEIN
7MXBA	SERINE/THREONINE PROTEIN KINASES
5DFZB	VACUOLAR PROTEIN SORTING ASSOCIATED PROTEIN
8FO9F	LEUCINE RICH REPEAT SERINE/THREONINE PROTEIN KINASE
2PZIA	PROBABLE SERINE/THREONINE PROTEIN KINASE PKNG
6O8BB	STIMULATOR OF INTERFERON GENES PROTEIN
8JPBG	BETA ADRENERGIC RECEPTOR KINASE
8SAMA	CLASS III LANTHIPEPTIDE, CLASS III LANTHIONINE
4OAUC	25 ADEPENDENT RIBONUCLEASE
5CE3B	ACTIN
3VWAA	CYTOPLASMIC EXPORT PROTEIN 1
3PS5A	TYROSINE PROTEIN PHOSPHATASE NONRECEPTOR TYPE 6
7Z3JA	1 PHOSPHATIDYLINOSITOL 4,5 BISPHOSPHATE PHOSPHODI
7T8TA	1 PHOSPHATIDYLINOSITOL 4,5 BISPHOSPHATE PHOSPHODI
8GWEA	RNA-DIRECTED RNA POLYMERASE
6P63C	SIDEROPHORE SYNTHETASE TYPE C
8SSNB	TYROSINE PROTEIN KINASE ABL
5EJRA	MYOSIN I HEAVY CHAIN
3JBZA	SERINE/THREONINE PROTEIN KINASE MTOR
3TO3B	PETROBACTIN BIOSYNTHESIS PROTEIN ASBB
3AU4A	MYOSIN X
3PVLA	MYOSIN VIIA ISOFORM 1
4YKNA	PHOSPHATIDYLINOSITOL 3 KINASE REGULATORY SUBUNIT
5XPYA	FERMITIN FAMILY HOMOLOG 2
8H7GC	TRANSFORMATION/TRANSCRIPTION DOMAIN ASSOCIATED PR
6QLYA	E3 UBIQUITINPROTEIN LIGASE MYLIP

PDB ID	Protein name
1YVLB	SIGNAL TRANSDUCER AND ACTIVATOR OF TRANSCRIPTION
7CT1A	FUSION PROTEIN SORTING NEXIN27 AND DLF MOTIF
7BI2A	PHOSPHATIDYLINOSITOL 4-PHOSPHATE 3-KINASE
3ZNIA	E3 UBIQUITINPROTEIN LIGASE CBLB
6FSFA	GTPASE-ACTIVATING PROTEIN BEM3
3CXLA	NCHIMERIN
6WLZY	V-TYPE PROTON ATPASE CATALYTIC SUBUNIT A
6O9L1	DNA-DIRECTED RNA POLYMERASE II SUBUNIT RPB1
6LK8J	MGC83295 PROTEIN
6PLMB	SIDJ PROTEIN
6ST5A	LICM2
6NF1A	PROTO ONCOGENE
4GZUA	FERM, RHOGEF, AND PLECKSTRIN DOMAIN CONTAINING
4XOHB	DIVISION MAL FOUTUE 1 PROTEIN
3TVVB	HISTONE CHAPERONE RTT106
7VS3A	CALCIUM DEPENDENT SECRETION ACTIVATOR 1
7C3MA	FERMITIN FAMILY HOMOLOG 3, FERMITIN FAMILY HOMOLOG
7QE7I	ANAPHASE PROMOTING COMPLEX SUBUNIT 10
8R33A	PHO90 ISOFORM 1
2KR0A	PROTEASOMAL UBIQUITIN RECEPTOR ADRM1
6T1ZA	LMRP INTEGRAL MEMBRANE PROTEIN
6ZU0C	CITRATE SYNTHASE
7VMBA	IQ MOTIF AND SEC7 DOMAIN-CONTAINING PROTEIN 1
8BTJA	PROTEIN M35
4BTFA	MIXED LINEAGE KINASE DOMAIN-LIKE PROTEIN
