# Supplementary material for: TKL3 regulates blood-stage fitness, male gamete fertility, and transmission-stage development in Plasmodium berghei
Source: PLoS Pathog. 2026 Sep 8;22(9):e1014582. doi: 10.1371/journal.ppat.1014582 (PMC13577473; doi:10.1371/journal.ppat.1014582)
Supplement: Supplementary file 2 [file ppat.1014582.s009.pdf]

S2 Table. List of primers used in this study.

Primer	Sequence
1215	GTTGTCTCTTCAATGATTCATAAATAG
1225	TTCCGCAATTTGTTGTACATA
1392	GCCCTCCATGTGCACCT
1583	CTCGAGGAGGTTTTATTTTTACATTATCT
1584	ATCGATTCATCTTTTTAATAAACTTAAGTT
1585	GCGGCCGCGATGGATGAAGTTAAGAAATAA
1586	GGCGCGCCGATACATAAGTTGTATTCATAC
1625	TCTATTGTGGAGATACAAGTT
1626	TGTTTGCCGCATGTTGCTT
1652	TGATAGTCCAAGTGCATACG
2053	CGGGCCCCCCCTCGAGCATCGTGATATTAAATTGTCTAAT
2054	CGTATGGGTAAGATCTTCCATCTAAAAAGGAAATCATATCA
2055	GTACCGACCGCGGCCGCTGAAGTTAAGAAATAAAACGCATG
2056	ATTACGCCAGGCGCGCCACATAAGTTGTATTCATACATGAT
2071	GTAGTTTACGACAAGGTGAAGA
2444	AACCATACGATTTAGTATATACC
