# Supplementary material for: TKL3 regulates blood-stage fitness, male gamete fertility, and transmission-stage development in Plasmodium berghei
Source: PLoS Pathog. 2026 Sep 8;22(9):e1014582. doi: 10.1371/journal.ppat.1014582 (PMC13577473; doi:10.1371/journal.ppat.1014582)
Supplement: Supplementary file 3 [file ppat.1014582.s011.pdf]

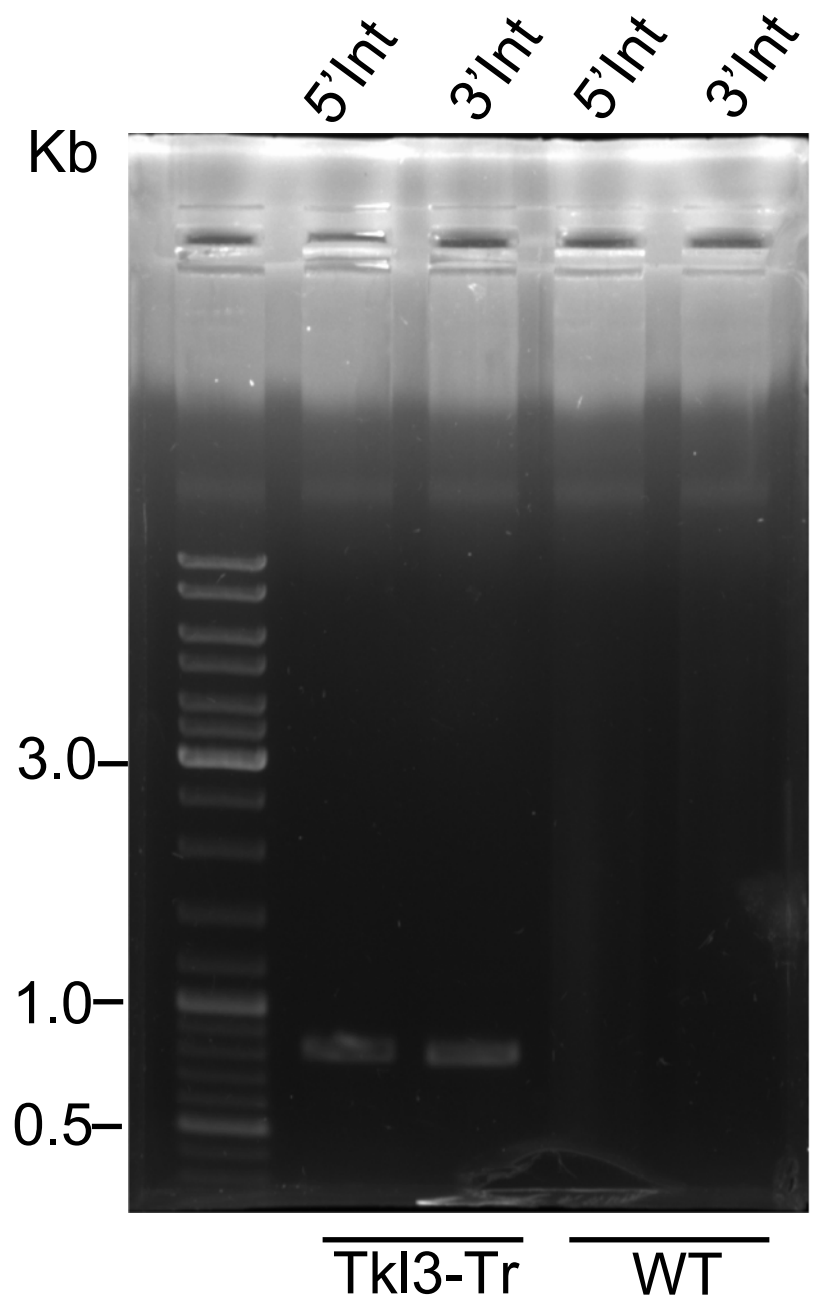

Un-cropped Image used to generate S3B Fig.

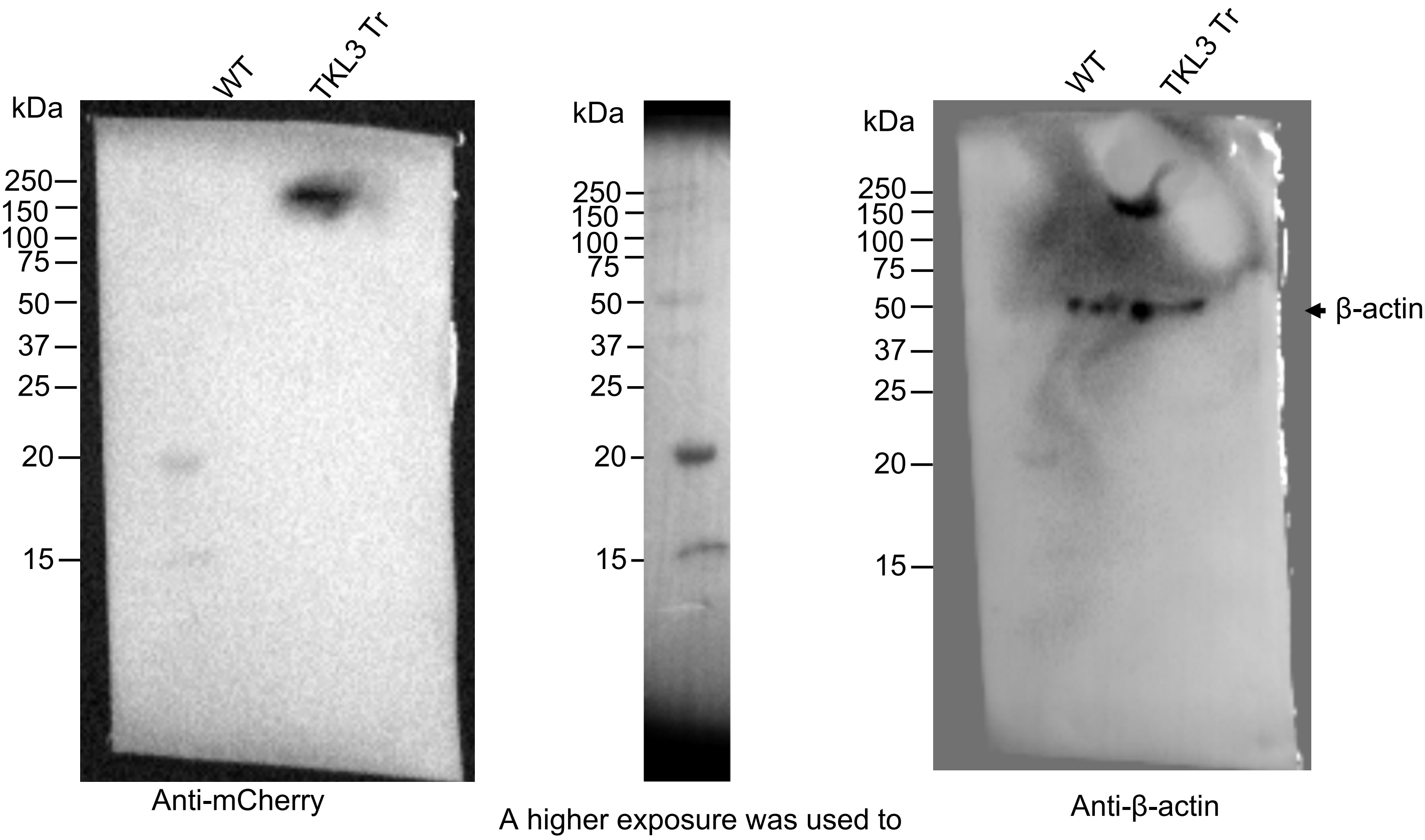

A higher exposure was used to visualize all the molecular weight ladder

Un-cropped Images used to generate S3F Fig.

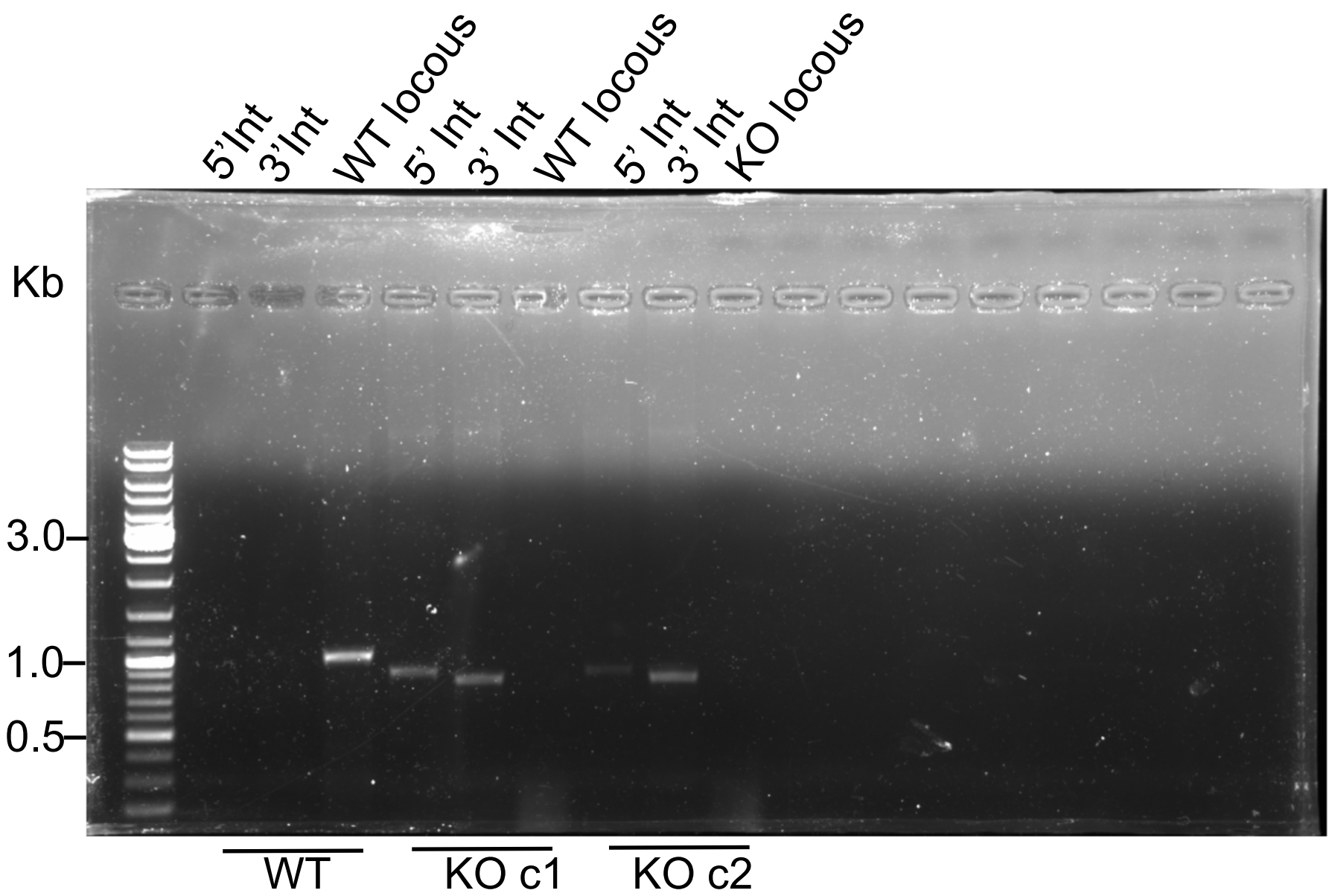

Un-cropped Image used to generate S6C Fig.

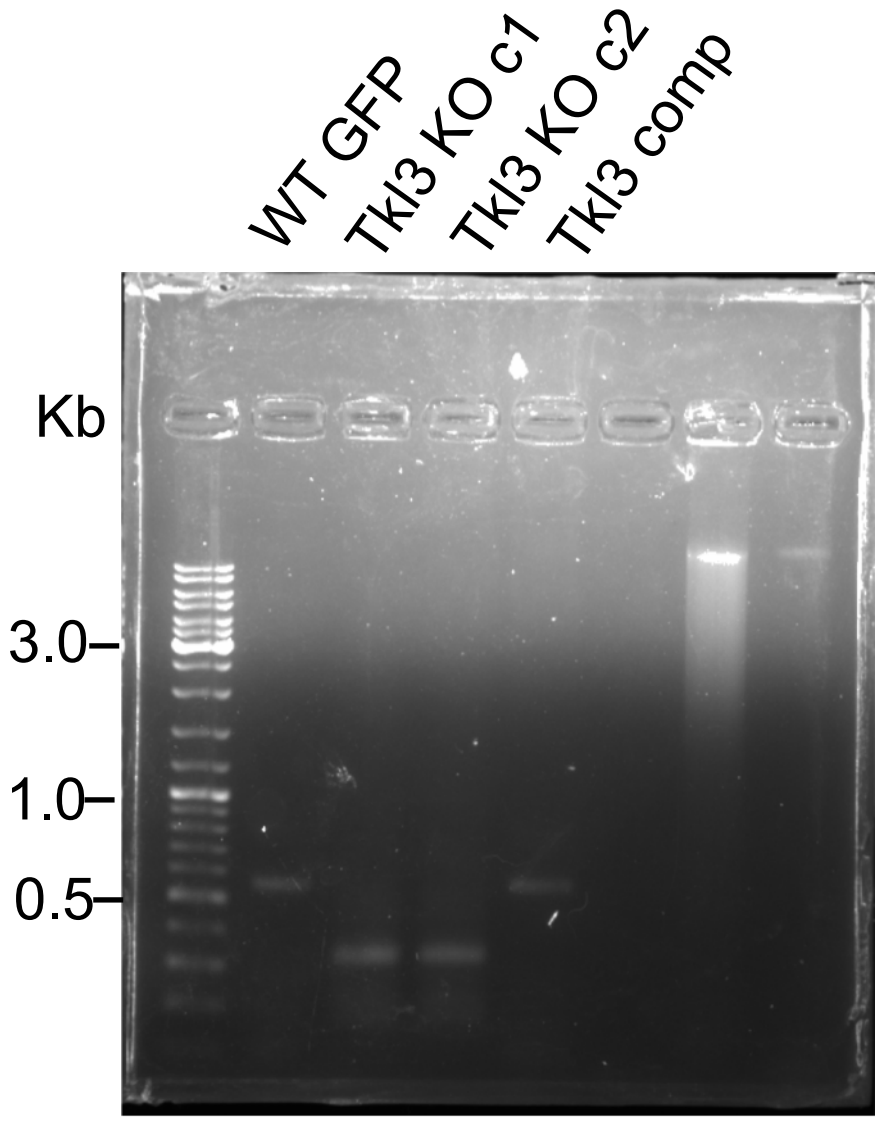

Un-cropped Image used to generate S6E Fig.
